# Supplementary material for: Exploratory Role of Flavonoids on Metabolic Dysfunction-Associated Steatotic Liver Disease (MASLD) in a South Italian Cohort
Source: Antioxidants (Basel). 2024 Oct 24;13(11):1286. doi: 10.3390/antiox13111286 (PMC11591465; doi:10.3390/antiox13111286)
Supplement: Supplementary file 1 [file antioxidants-13-01286-s001.zip › antioxidants-3213769-supplementary.pdf]

Table S1. MASLD No and MASLD Yes groups according to the positive diagnostic criteria

Criteria MASLD	MASLD No	MASLD Yes
N	668 (51.50%)	629 (48.50%)
Hepatic Steatosis		
No	668 (100.0%)	0 (0.0%)
Yes	0 (0.0%)	629 (100.0%)
BMI ≥ 25 kg/m^2	329 (37.8%)	542 (62.2%)
Waist: M > 94 cm and F >80 cm	270 (33.4%)	539 (66.6%)
SBP ≥ 130 (mmHg)	170 (34.6%)	321 (65.4%)
DBP ≥85 (mmHg)	668 (51.5%)	629 (48.5%)
Serum Glucose ≥ 100 mg/dL	96 (27.4%)	255 (72.6%)
Triglycerides ≥ 150 mg/dL	57 (25.9%)	163 (74.1%)
HDL cholesterol < 40 mg/dL for M and < 50 mg/dL for F	192 (42.7%)	258 (57.3%)

MASLD: Metabolic dysfunction-associated steatotic liver disease; BMI: Body Mass Index; MASLD: Metabolic dysfunction-associated steatotic liver disease; HDL-C: High-Density Lipoprotein Cholesterol.

Table S2. Amount mean (±SD) of flavonoids (mg/day) contained in the following health foods by MASLD in the Nutrihep cohort.

	MASLD		*p-value*[¥]
	No	Yes	
N	668	629	
Flavonoids	189.63 (112.85)	211.81 (113.54)	<0.001
Apples	85.80 (87.33)	101.03 (85.57)	0.002
Peaches	22.84 (18.13)	23.56 (17.82)	0.47
Pear	19.37 (20.92)	23.79 (25.85)	<0.001
Strawberries	17.15 (28.44)	17.62 (31.73)	0.78
Oranges	16.27 (16.35)	18.05 (13.31)	0.031
Grapes	14.88 (16.23)	15.85 (17.17)	0.29
Walnuts	6.10 (9.96)	6.45 (9.57)	0.52
Orange Juice	4.13 (10.15)	4.10 (12.50)	0.96
Spinach	1.41 (1.73)	1.32 (1.67)	0.38
Courgettes	0.98 (1.05)	0.88 (1.03)	0.090
Broccoli	0.91 (0.89)	0.98 (1.02)	0.21
Cooked onion	0.68 (1.42)	0.74 (1.46)	0.47
Aubergines	0.64 (0.68)	0.58 (0.69)	0.17
Tomatoes	0.54 (0.50)	0.55 (0.48)	0.61
Celery	0.45 (0.49)	0.43 (0.49)	0.55
Onions	0.17 (0.39)	0.25 (0.50)	<0.001

[¥] Wilcoxon rank-sum. MASLD: Metabolic dysfunction-associated steatotic liver disease

Table S3 . Lasso selects covariates logit model

Selection: Bayesian information criterion

ID	Description	lambda	No. of nonzero coef.	In-sample dev. ratio	BIC
1	first lambda	.1679354	3	0.0202	1782.149
31	lambda before	.0103044	10	0.2429	1433.747
* 32	selected lambda	.0093889	10	0.2443	1431.251
33	lambda after	.0085549	11	0.2455	1436.268
74	last lambda	.0001886	14	0.2547	1441.344

* lambda selected by Bayesian information criterion.

	minBIC	adaptive	cv
weight	x	x	x
age65	x	x	x
γGT	x	x	x
sex_1M	x	x	x
ALT	x	x	x
flavonoidis	x	x	x
kcal_day	x	x	x
Marital_Status	x	x	x
homa2_5	x	x	x
job	x	x	x
_cons	x	x	x

Table S4. Collinearity Diagnostics by VIF for the independent variables

Indipendent Variables	VIF	SQRT VIF	Tolerance	R^2
Flavonoids (mg/day)	1.16	1.08	0.8589	0.1411
Job	1.39	1.18	0.7211	0.2789
Kcal (day)	1.23	1.11	0.8130	0.1870
Marital Status	1.10	1.05	0.9084	0.0916
ALT	1.41	1.19	0.7117	0.2883
γGT	1.46	1.21	0.6862	0.3138
Gender	1.38	1.18	0.7224	0.2776
Age (<65 yrs vs ≥65 yrs)	1.57	1.25	0.6367	0.3633
Homa (<2.5 vs ≥2.5)	1.13	1.06	0.8883	0.1117
Weight (kg)	1.41	1.19	0.7087	0.2913
Mean VIF	1.32			

VIF: Variance Inflation Factors; ALT: Alanine Aminotransferase; γGT: Gamma Glutamyl Transferase; HOMA: Homeostasis Model Assessment.

Table S5. Characteristics of Participants by Median Value of Flavonoids in the Nutrihep cohort.

Parameters [a]	Flavonoids (mg/day)	
	<185	≥185
N	648	649
Age (years)	50.71 (13.94)	57.94 (13.82)
Age categories (years) (%)		
<65	521 (54.6)	434 (45.4)
≥65	127 (37.1)	215 (62.9)
Gender (%)		
Female	379 (50.9)	365 (49.1)
Male	269 (48.6)	284 (51.4)
MASLD (%)		
No	367 (54.9)	301 (45.1)
Yes	281 (44.7)	348 (55.3)
Flavonoids (mg/day)	110.97 (46.54)	289.67 (87.79)
SBP (mmHg)	118,66 (15,40)	123,18 (15,90)
DBP (mmHg)	77,07 (8,20)	78,28 (7,75)
rMED (median (IQR))	7.00 (6.00-9.00)	9.00 (7.00-10.00)
BMI (kg/m^2)	27.23 (5.26)	27.94 (4.80)
Weight (kg)	72.65 (15.56)	73.20 (14.16)
Waist (cm)	89.19 (13.90)	91.70 (12.90)
HbA1c (mmol/mol)	37.59 (6.38)	38.54 (7.31)
HOMA	1.87 (1.82)	1.90 (1.94)
ALT (U/L)	22.85 (20.96)	21.55 (9.29)
ɣGT (U/L)	17.75 (15.11)	17.41 (11.58)
AST (U/L)	21.87 (14.39)	21.61 (5.46)
TG (mg/dL)	92.70 (64.27)	104.08 (73.45)
C-Reactive Protein (mg /dL)	0.26 (0.65)	0.25 (0.43)
TC (mg/dL)	190.95 (34.26)	191.75 (36.44)
HDL (mg/dL)	51.25 (12.82)	50.33 (12.35)
Glucose (mg/dL)	93.61 (17.04)	97.05 (17.48)
ALP (U/L)	52.52 (17.60)	53.44 (14.44)
Alcohol intake (g/day)	10.96 (13.38)	10.21 (12.03)
Kcal (day)	1901.92 (708.62)	2210.37 (759.39)
Smoker (%)		
Never/Former	557 (49.0)	580 (51.0)
Current	90 (56.6)	69 (43.4)
Hypertension (%)		
No	459 (54.2)	388 (45.8)
Yes	157 (40.8)	228 (59.2)
Dyslipidemia (%)		
No	546 (52.1)	501 (47.9)
Yes	69 (37.5)	115 (62.5)
Diabetes (%)		

No	585 (51.0)	563 (49.0)
Yes	31 (36.9)	53 (63.1)

[a]As Means and Standard Deviation. MASLD: Metabolic dysfunction-associated steatotic liver disease; SBP: Systolic Blood Pressure; DBP: Diastolic Blood Pressure; rMED: Relative Mediterranean Diet; BMI: Body Mass Index; HbA1c: Glycosylated Haemoglobin; HOMA: Homeostasis Model Assessment; ALT: Alanine Aminotransferase; ɣGT: Gamma Glutamil Transferase; AST: Aspartate Aminotransferase; TG: Triglycerides; TC: Total Cholesterol; HDL-C: High-Density Lipoprotein Cholesterol; ALP: Alkaline Phosphatase Level.

Table S6. Absolute frequencies on deciles of daily flavonoid.

**Flavonoid intake (mg/day)**	**0-10 (2; 66)**	**10-20 (66; 97)**	**20-30 (97; 129.4)**	**30-40 (129.4 155)**	**40-50 (155; 185)**	**50-60 (185; 211.8)**	**60-70 (211.8; 245.6)**	**70-80 (245.6; 290.4)**	**80-90 (290.4; 354.2)**	**90-100 (354.2; 500)**
**NO MASLD**	81	78	70	70	68	62	54	58	64	61
**MASLD**	50	50	58	64	59	63	75	71	65	68
**SUM**	131	128	128	134	127	125	129	129	129	129

Table S7. Logistic regression analysis of MASLD on flavonoid intake divided into deciles

	p-value	95% CI
OR_{d2} = 1.258	0.468	0.675; 2.345
OR_{d3} = 1.571	0.155	0.842; 2.931
OR_{d4} = 1.733	0.078	0.939; 3.199
OR_{d5} = 1.819	0.064	0.964; 3.434
OR_{d6} = 2.173	0.014	1.166; 4.053
OR_{d7} = 2.451	0.004	1.317; 4.563
OR_{d8} = 2.316	0.008	1.239; 4.329
OR_{d9} = 1.625	0.133	0.861; 3.066
OR_{d10} = 1.951	0.039	1.034; 3.681

ref. d1: 1st decile. Models adjusted for Job, Daily calories, Weight (kg), Gamma Glutamyl Transferase, Alanine Aminotransferase, gender (Female vs Male), age (<65 vs ≥65 years), Marital status, Homa (<2.5 vs ≥2.5)
